# Multiplex real-time quantitative PCR, microscopy and rapid diagnostic immuno-chromatographic tests for the detection of *Plasmodium spp*: performance, limit of detection analysis and quality assurance

**DOI:** 10.1186/1475-2875-8-284

**Published:** 2009-12-09

**Authors:** Krishna Khairnar, Donald Martin, Rachel Lau, Filip Ralevski, Dylan R Pillai

**Affiliations:** 1 Department of Laboratory Medicine & Pathobiology, University of Toronto, 81A Resources Road, Rm 243, Toronto ON M9P 3T1, ON, Canada; 2 Department of Medicine, University of Toronto, ON, Canada; 3 Ontario Agency for Health Protection and Promotion, Toronto, ON, Canada

## Abstract

**Background:**

Accurate laboratory diagnosis of malaria species in returning travelers is paramount in the treatment of this potentially fatal infectious disease.

**Materials and methods:**

A total of 466 blood specimens from returning travelers to Africa, Asia, and South/Central America with suspected malaria infection were collected between 2007 and 2009 at the reference public health laboratory. These specimens were assessed by reference microscopy, multipex real-time quantitative polymerase chain reaction (QPCR), and two rapid diagnostic immuno-chromatographic tests (ICT) in a blinded manner. Key clinical laboratory parameters such as limit of detection (LOD) analysis on clinical specimens by parasite stage, inter-reader variability of ICTs, staffing implications, quality assurance and cost analysis were evaluated.

**Results:**

QPCR is the most analytically sensitive method (sensitivity 99.41%), followed by CARESTART (sensitivity 88.24%), and BINAXNOW (sensitivity 86.47%) for the diagnosis of malaria in returning travelers when compared to reference microscopy. However, microscopy was unable to specifically identify *Plasmodia spp*. in 18 out of 170 positive samples by QPCR. Moreover, the 17 samples that were negative by microscopy and positive by QPCR were also positive by ICTs. Quality assurance was achieved for QPCR by exchanging a blinded proficiency panel with another reference laboratory. The Kappa value of inter-reader variability among three readers for BINAXNOW and CARESTART was calculated to be 0.872 and 0.898 respectively. Serial dilution studies demonstrated that the QPCR cycle threshold correlates linearly with parasitemia (R^2 ^= 0.9746) in a clinically relevant dynamic range and retains a LOD of 11 rDNA copies/μl for *P. falciparum*, which was several log lower than reference microscopy and ICTs. LOD for QPCR is affected not only by parasitemia but the parasite stage distribution of each clinical specimen. QPCR was approximately 6-fold more costly than reference microscopy.

**Discussion:**

These data suggest that multiplex QPCR although more costly confers a significant diagnostic advantage in terms of LOD compared to reference microscopy and ICTs for all four species. Quality assurance of QPCR is essential to the maintenance of proficiency in the clinical laboratory. ICTs showed good concordance between readers however lacked sensitivity for non-*falciparum *species due to antigenic differences and low parasitemia.

**Conclusion:**

Multiplex QPCR but not ICTs is an essential adjunct to microscopy in the reference laboratory detection of malaria species specifically due to the superior LOD. ICTs are better suited to the non-reference laboratory where lower specimen volumes challenge microscopy proficiency in the non-endemic setting.

## Background

The protozoan parasite *Plasmodium *which causes malaria is a vector-borne infectious disease, estimated to infect approximately 350-500 million and kill more than a million people world wide every year [1]. There are five species of the *Plasmodium *parasite that can infect humans: the most virulent form of the disease is caused by *Plasmodium falciparum *and *Plasmodium knowlesi*. Malaria caused by *Plasmodium vivax*, *Plasmodium ovale *and *Plasmodium malariae*, are more chronic disease in humans. In the past three decades, with the globalization of economy and increasing immigration frequency, there has been a significant rise in the number of cases of imported malaria in non-endemic, developed countries, such as Canada [2]. Toronto is a large urban center in Canada with high rates of international travel and immigration and has recently been subjected to waves of imported outbreaks, such as SARS and swine H1N1 influenza [3].

Microscopic identification of malaria parasites relies on examination of Giemsa-stained peripheral blood smears. The sensitivity and specificity of this method are highly affected by the staining techniques used and the skill level of the microscopist [4-9]. Although light microscopy is capable of defining parasite density, parasite stage, and speciation, this method is labour-intensive and requires well-trained experts at reference centres, not to mention delays in specimen transport to such centres. In order to make the diagnosis of malaria faster and simpler to perform, rapid tests have been developed based on antigen-capture immuno-chromatographic tests (ICTs) as a valuable adjunct to microscopy for the diagnosis of malaria [2]. The ICTs are useful for identifying *P. falciparum *infection, but cannot be used to specifically identify non-*P. falciparum *species infections such as *P. vivax*, *P. ovale*, and *P. malariae *[7].

In the past few decades, with the advent of technology in molecular biology, new molecular methods have been introduced, such as polymerase chain reaction (PCR) and real-time quantitative polymerase chain reaction (QPCR). These molecular methods can detect malaria parasites to the species level by targeting the parasite DNA with good analytical sensitivity and specificity in laboratory studies [2,10-13]. QPCR in particular has been a successful method for microbial detection from sterile sites with excellent sensitivity and has the ability to be quantitative. Many of the studies conducted to date seeking to validate malaria molecular diagnostics are limited to the research laboratory, but reports emphasizing the implementation of these techniques in clinical laboratory setup have not been adequately investigated in the past [14]. In the present study, validation of QPCR as compared to reference microscopy and two different ICTs was performed with particular attention to evaluating limits of detection, quality assurance, cost, staffing, and implementation in the clinical microbiology laboratory.

## Materials and methods

### Sample details

A total of 466 blood samples from individual patients (returning travelers) with fever or a history of fever and travel to a malaria-endemic area were tested by standard Giemsa stain microscopy for *Plasmodium *species detection. Summary statistics of patient clinical specimens are summarized in Table 1. Blood specimens were collected at community and hospital laboratories in Ontario, Canada and forwarded by courier the same day to the Public Health Reference Laboratory in Toronto (PHL Toronto) during the period May 2007 to January 2009. Infected blood aliquots were banked in a malaria bio-repository (-80°C monitored freezer) at PHL Toronto. Blood specimens positive by microscopy as well as a subset negative by microscopy were included in this study. Microscopy, ICTs (BINAXNOW^® ^MALARIA [Binax, Seattle, WA] and CARESTART™ [Accessbio, Monmouth Junction, NJ]) detection and QPCR were performed in a blinded manner.

**Table 1:** Summary of clinical specimens used in this study.

Patient characteristic	Value
Male (%)	55.98

Female (%)	44.02

Average Age	31.16

Age range	1-90

Geometric Mean Parasitaemia (%)	0.095

Previous travel to Africa (%)	28.5

Previous travel to Asia (%)	42.3

Previous travel to Americas (%)	29.2

### Conventional microscopy

Microscopy of thick and thin Giemsa-stained smears was performed according to standard methods by at least two experienced microscopists for each specimen reported here [15]. The parasitaemia is expressed as a percentage of erythroyctes. Microscopy performed here is considered reference microscopy and undergoes external quality assurance as a participant of the Insitut National de Santé Publique du Québec (INSPQ) blood parasitology programme.

### Immuno-chromatographic tests (ICT)

Two rapid ICTs (BINAXNOW^® ^MALARIA [Binax, Seattle, WA] and CARESTART ™, [Accessbio, Monmouth Junction, NJ]) were used in this study to detect the circulating *Plasmodium *antigen in the whole blood for the diagnosis of malaria. Both of the ICTs detect the *Plasmodium falciparum*-specific HRP2 antigen as well as a panmalarial antigen common to all *Plasmodium *species (aldolase in the case of BINAXNOW and pLDH in the case of CARESTART). The assays were performed according to the manufacturer's instructions. The test results were independently examined and interpreted by three blinded observers (one of whom was not a trained medical laboratory technologist). The final results of the test were recorded as either negative or positive based on the majority agreement. While ICT results reported here were on fresh frozen specimens,, all specimens were frozen only once, kept under monitored refrigerator conditions according to Clinical Laboratory Improvement Amendments (CLIA) guidelines. No difference was seen between single frozen and fresh frozen specimens when tested by ICT.

### Multiplex real-time quantitative polymerase chain reaction (QPCR)

DNA was extracted from 100 μl of thawed EDTA-whole blood using the QIAamp DNA Mini Kit (Qiagen, Valencia, CA) according to the manufacturer's instructions. The extracted DNA was stored at -20°C in sterile capped containers until used. The qPCR was performed by using primers and reaction conditions described previously by Alberta Public Health Laboratory with the following modifications: fluorophores for probes of *P. facliparum *were changed to FAM-MGBNFQ and *P. vivax *to VIC-MGBNFQ [12]. Briefly, qPCR was performed under standard conditions (1 cycle of 50°C for 2 mins; 1 cycle of 95°C for 10 mins; 45 cycles of 95°C for 15 s and 60°C for 1 min) with the ABI TaqMan 7900 (Applied Biosystems Inc, Foster City, CA). The reaction was performed with a final volume of 25 μl containing 5 μl of template DNA, 12.5 μl of TaqMan universal master mix (ABI), and 7.5 μl of pooled primers and probes mix. Samples were confirmed *Plasmodium *positive with genus-specific primers Plasmo1 and Plasmo2 and the Plasprobe to detect a region of the *Plasmodium *18S gene that is conserved across all five species. The *Plasmodium *species present in the sample were determined with species-specific forward primers, Plasmo2 and species-specific probes. The reaction was performed by multiplexing two *plasmodium *species in a single tube with distinct fluorophores for each probe, e.g. *P. falciparum*/*P. vivax *in a one tube and *P. malariae*/*P. ovale *in another tube. A cutoff of 40 cycles was used to define positive samples [12]. The test panel also included a number of controls: negative sample extraction as a negative control, β2-macroglobulin target amplification as a positive extraction control for the specimen, a positive reference control to detect any variation between runs, and no template negative controls for each of the master mixes.

### Quality assurance

QPCR results were also compared with another reference provincial laboratory for public health, Edmonton, Alberta, Canada, using a blinded panel of 10 randomly selected blood specimens as part of quality assurance. The 10 blinded panel of blood specimens included *P. vivax *(*n *= 3), *P. falciparum *(*n *= 3), *P. ovale *(*n *= 3), mixed infection of *P. falciparum *and *P. malariae *(*n *= 1), and negative (*n *= 1). Microscopists used in this study are part of the INSPQ blood parasitology, quality assurance programme.

### Limit of detection (LOD) for clinical isolates

To determine the minimum number of *Plasmodium *parasites detectable by two ICTs and QPCR, blood samples from twelve patients infected with *P. falciparum *(*n *= 3), *P. vivax *(*n *= 3), *P. malariae *(*n *= 3), and *P. ovale *(*n *= 3) were collected. The DNA purified from serial dilutions was performed in triplicate by QPCR. The ICTs were performed in parallel on all the blood sample serial dilutions, the results were interpreted by two different readers. The microscopy on serial dilutions was ruled out because the blood samples were already stored at -20°C in the malaria bio-repository. In addition, a LOD study was also done prospectively from three patients who from the Democratic Republic of Congo and were positive for *P. falciparum *by reference microscopy. To establish the ability of assays like ICT and QPCR to detect only the gametocyte stage of *Plasmodium *parasite, blood sample from one patient exclusively infected with *P. falciparum *gametocyte stage was used. All the blood samples were subjected to serial dilution with uninfected erythrocytes from healthy individuals with known baseline erythrocyte counts. The DNA purified from the dilutions was treated in triplicates for QPCR assay.

### Limit of detection (LOD) for laboratory strains

The *P. falciparum *3D7 strain was grown in two sets in human Rh^+ ^erythrocytes at a haematocrit level of 5% in complete RPMI 1640 medium in tissue culture flasks (25 cm^2 ^and 75 cm^2^) according to the method of Trager and Jensen [16]. The culture flask was replenished with a fresh batch of medium every 24 hours, and the culture was routinely monitored through Giemsa-staining of thin smears. The *P. falciparum *culture was synchronized to get only ring stages of *P. falciparum *as described earlier using 5% D-sorbitol [17]. The parasitaemia in the synchronized culture was determined from Giemsa-stained thin smears. In these malaria cultures to express the parasitaemia as number of parasites present in 1 μl of culture, the RBC/μl was counted using a standard Neubauer haemocytometer as per the manufacturer's instructions. The two sets of synchronized cultures were subjected to tenfold serial dilution with uninfected erythrocytes from healthy individuals. The DNA purified from the dilutions was performed in triplicates for QPCR assay. The microscopy and ICTs were performed in parallel on all the serial dilutions, the results were interpreted by two different readers.

### Quantitating parasitaemia and ribosomal (r) DNA copy number from frozen specimens, fresh isolates, and laboratory strains

In frozen blood specimens to express the parasitaemia as number of parasites present in 1 μl of blood, it was assumed that 1 μl of blood contained 5 × 10^6 ^red blood cells, so 1% parasitaemia corresponds to 1 parasitized red blood cell/100 red blood cells or 50,000 parasites/μl of blood [18]. In the three fresh blood specimens to express the parasitaemia as number of parasites present in 1 μl of blood, the RBC/μl was calculated using a standard Neubauer haemocytometer as per the manufacturer's instructions. Infected blood samples were diluted with uninfected erythrocytes from healthy individuals with known baseline erythrocyte counts. Tenfold serial dilutions were made for each blood sample. The stage specific parasitaemia proportion was identified by a microscopist into different stages, such as single ring, double rings, triple rings, growing trophozoites, immature trophozoites, mature trophozoites, gametocytes, mature schizonts, and immature schizonts. The QPCR was performed on the ten-fold dilutions of these blood samples.

The rDNA copy number was calculated by using the following formula: rDNA copy number/reaction = (number. of parasitized RBC/μl)* ploidy * genecopy *5. The actual parasite ploidy number was determined as describes elsewhere [19]. Briefly, the ploidy for various stage of malarial parasites like, single ring and growing trophozoites was 1; double ring and gametocytes was 2; immature trophozoite, mature schizont and immature schizont was 8; and mature trophozoite was 16. The average rDNA gene copy number per parasite was considered as 6. A factor of 5 was multiplied, which accounts for the loading volume of DNA template per reaction.

### Statistical data analysis

Sensitivity was calculated as follows: number of true positives divided by number of true positives plus false negatives × 100. Specificity was calculated as follows: number of true negatives divided by the number of true negatives plus false positives × 100. Separate analyses were conducted using reference microscopy and QPCR as gold standards. To determine the statistical significance of the results, the confidence interval (CI) and the margin of error for the sample size were determined [20]. The inter-reader variability was calculated by applying Kappa statistic [21].

## Results

### Comparison of microscopy, ICTs, and RTPCR

Evaluation of the sensitivity and specificity of microscopy, BINAXNOW, and CARESTART using QPCR and microscopy separately as the gold standard was carried out (Table 2). The microscopy was found to have higher sensitivity and specificity than the ICTs. The sensitivity of CARESTART (85.2%) was higher than BINAXNOW (83.67%), and both the ICTs had similar specificities (99.26%). The discordant results of microscopy, ICTs, and QPCR are summarized in Table 3. Among 466 patients in whom malaria was suspected, 170 (36.5%) were positive by microscopy. The remaining 296 samples were negative. Microscopy was used as the reference standard for comparison with the other methods. The CI for the sample size of 466 was 95% and the margin of error was 4.49%. The BINAXNOW detected malaria parasites in 166 of 170 microscopically positive samples with a sensitivity of 86.47% (95% confidence interval [CI] 81.98-90.96). The CARESTART detected malaria parasites in 169 of 170 microscopically positive samples with a sensitivity of 88.24% (95% CI 83.75-92.73). Among the 296 samples that were negative by microscopy, both ICTs detected malaria parasites in 19 samples with a specificity of 93.58% (95% CI 89.09-98.07). The QPCR detected malaria parasites in 169 of 170 microscopically positive samples with a sensitivity of 99.41% (95% CI 94.92-100). The QPCR detected malaria parasites in 27 of 296 microscopically negative samples with a specificity of 90.88% (95% CI 86.39-95.37). Out of these 27 QPCR positive but microscopy negative samples, 17 were also positive by ICTs. Ten samples were exclusively positive by QPCR and negative by microscopy and ICTs. The QPCR was negative in one out of 170 microscopically positive samples; this sample was also negative by ICTs. The QPCR has highest sensitivity (99.41%) in comparison to BINAXNOW (86.47%) and CARESTART (88.24%).

**Table 2:** Sensitivity and specificity expresessed as perecentarge of microscopy and ICTs (BINAXNOW and CARESTART) as alternately compared to QPCR and microscopy as gold standard method.

Assay type	QPCR		Microscopy	
	
	Sensitivity	Specificity	Sensitivity	Specificity
Microscopy	86.22	99.63	NA*	NA

BINAXNOW	83.67	99.26	86.47	93.58

CARESTART	85.2	99.26	88.24	93.58

QPCR	NA	NA	99.41	90.88

* NA - Not Applicable

**Table 3:** Discrepant analysis of different assays for the laboratory diagnosis of malaria based on results of QPCR.

Species	Microscopy & BINAXNOW & CARESTART Positive	Microscopy & BINAXNOW & CARESTART Negative	Microscopy & BINAXNOW Positive	Microscopy & CARESTART Positive	BINAXNOW & CARESTART Positive	Only Microscopy Positive	Only BINAXNOW Positive	Only CARESTART Positive
***Pf***	85	4	1	2	16	2	0	1

***Pv***	54	5	0	3	0	10	0	0

***Pm***	1	1	0	0	0	1	0	0

***Po***	1	0	0	0	0	4	0	0

***Pf & Pv***	3	0	0	0	0	0	0	0

***Pm & Po***	0	0	1	0	0	0	0	0

***Pf & Pm***	1	0	0	0	0	0	0	0

**Negative**	0	266	0	0	2	1	1	0

**Total**	145	276	2	5	18	18	1	1

### Conventional microscopy

A total of 170 out of 466 blood samples screened were positive for *Plasmodium spp *by microscopy. These 170 microscopy positive included 82 positive for *P. falciparum*, 63 positive for *P. vivax*, one positive for *P. ovale*, two positive for *P. malariae*, two positive for *P. falciparum *+ *P. ovale *(mixed), one positive for *P. falciparum *+ *P. vivax *(mixed), one positive for *P. falciparum *+ *P. malariae *(mixed) and 18 positive for *Plasmodium spp *(species remained undetermined). 296 blood samples were negative for *Plasmodium spp *by microscopy. Microscopy was used as the reference standard for comparison with the other methods.

### Immuno-chromatographic tests (ICT)

The BINAXNOW was positive for *Plasmodium spp *in 35.6% (166 out of 466) blood samples. These 166 BINAXNOW positive included 84 positive for *P. falciparum*/mixed *Plasmodium *species, 23 positive for *P. falciparum*, 59 positive for non *P. falciparum *species. The CARESTART was positive for *Plasmodium spp *in 36.3% (169 out of 466) blood samples. These 169 CARESTART positive included 84 positive for *P. falciparum*/mixed *Plasmodium *species, 22 positive for *P. falciparum*, 63 positive for non *P. falciparum *species. The Kappa value of inter-reader variability among three readers for BINAXNOW and CARESTART was calculated to be 0.872 and 0.898 respectively.

### Multiplex-QPCR

The QPCR was positive for *Plasmodium spp *in 42.1% (196 out of 466) of blood samples. These 196 RTPCR positive included 111 positive for *P. falciparum*, 72 positive for *P. vivax*, five positive for *P. ovale*, three positive for *P. malariae*, one positive for *P. malariae *+ *P. ovale *(mixed), and three positive for *P. falciparum *+ *P. vivax *(mixed), one positive for *P. falciparum *+ *P. malariae *(mixed). The result of multicentre validation of RTPCR on a blinded panel of 10 randomly selected blood specimens demonstrated 100% concordance. A different blinded panel was forwarded to the Alberta provincial reference laboratory, where 100% concordance was also achieved. This process was repeated six months later with a different blinded panel with 100% agreement as part of an ongoing quality control procedure. However, the comparative results of the multicentre validation showed that the Ct values were slightly lower than that performed by Alberta (see Additional File 1). All four *Plasmodium *species were successfully differentially detected in the species specific multiplex QPCR reaction.

### Limit of detection analyses

**Figure 1:**
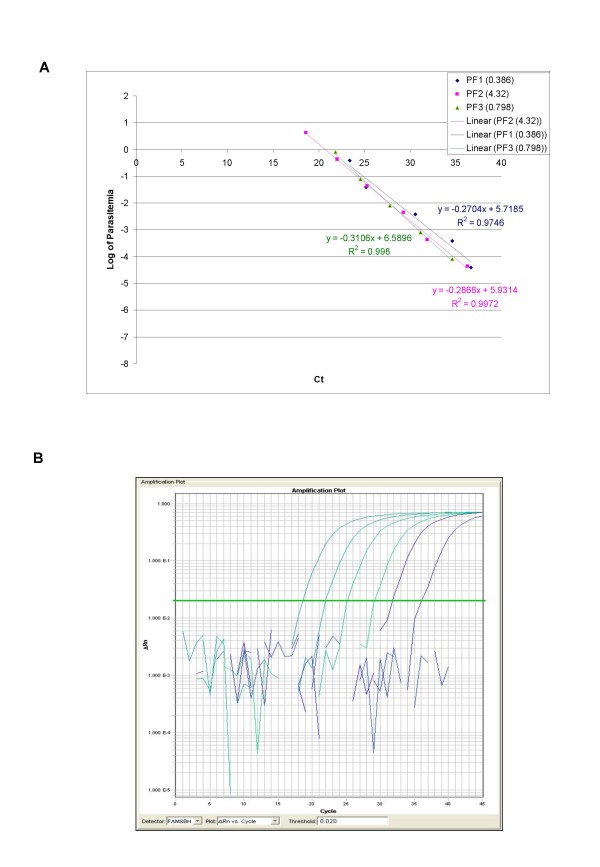
**A plot of log of parasitaemia versus threshold cycle for the 10-fold serial dilutions of *P. falciparum *(A) and a representative amplification plot for 10-fold serial dilutions (B) generated by QPCR are also shown**. All clinical isolates were amplified in triplicate for each species with the linear correlation coefficient (R^2^) shown in the corresponding color.

**Figure 2:**
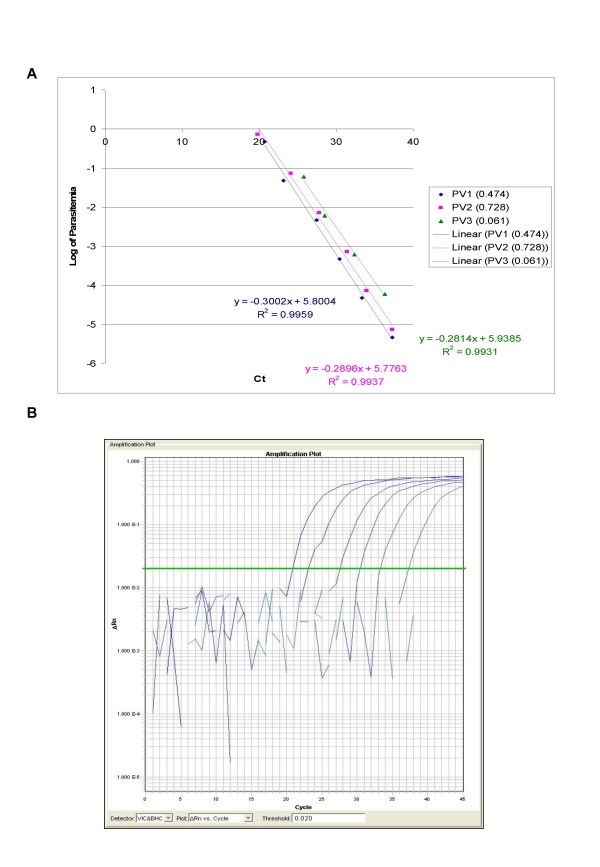
**A plot of log of parasitaemia versus threshold cycle for the 10-fold serial dilutions of *P. vivax *(A) and a representative amplification plot for 10-fold serial dilutions (B) generated by QPCR are also shown**. All clinical isolates were amplified in triplicate for each species with the linear correlation coefficient (R^2^) shown in the corresponding color.

**Figure 3:**
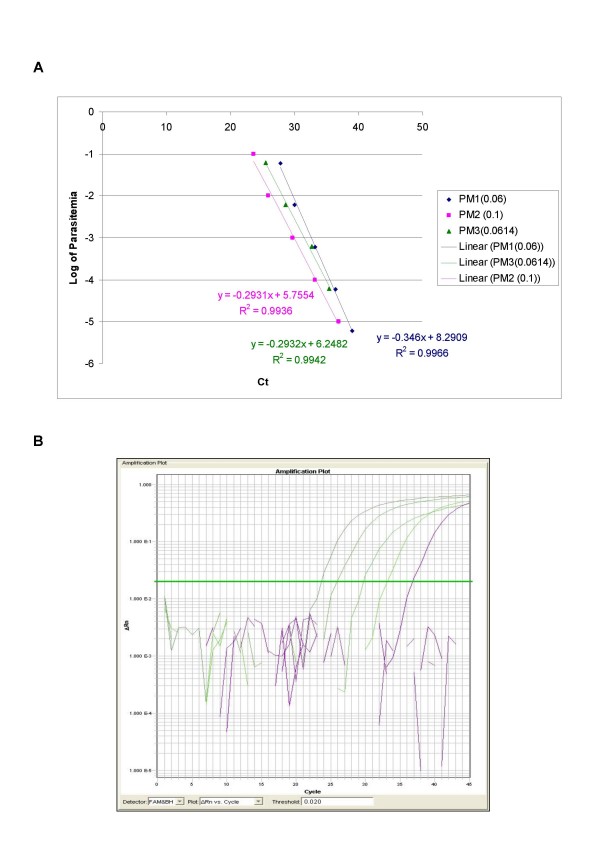
**A plot of log of parasitaemia versus threshold cycle for the 10-fold serial dilutions of *P. malariae *(A) and a representative amplification plot for 10-fold serial dilutions (B) generated by QPCR are also shown**. All clinical isolates were amplified in triplicate for each species with the linear correlation coefficient (R^2^) shown in the corresponding color.

**Figure 4:**
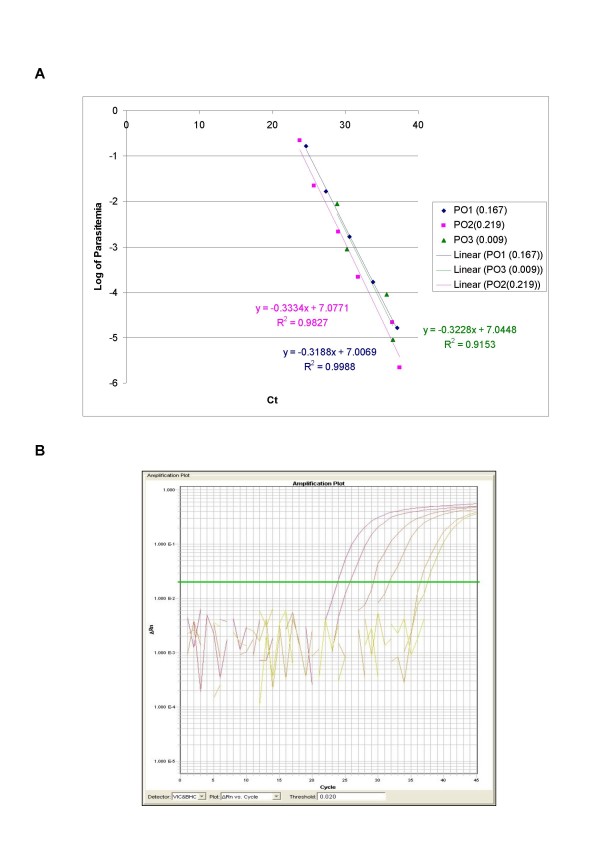
**A plot of log of parasitaemia versus threshold cycle for the 10-fold serial dilutions of *P. ovale *(A) and a representative amplification plot for 10-fold serial dilutions (B) generated by QPCR are also shown**. All clinical isolates were amplified in triplicate for each species with the linear correlation coefficient (R^2^) shown in the corresponding color.

**Figure 5:**
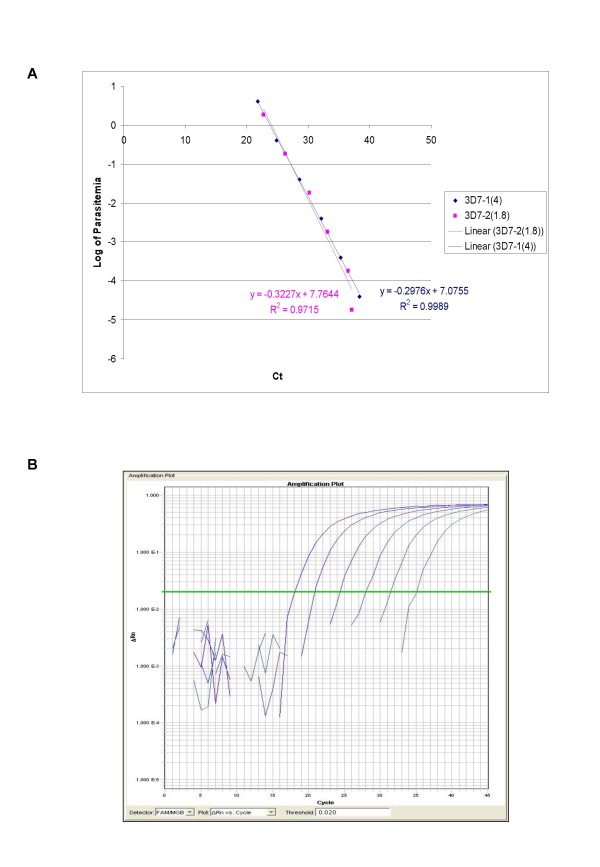
**A plot of log of parasitaemia versus threshold cycle for the 10-fold serial dilutions of *P. falciparum *laboratory strain 3D7 (A) and a representative amplification plot for 10-fold serial dilutions (B) generated by QPCR are also shown**. All clinical isolates were amplified in triplicate for each species with the linear correlation coefficient (R^2^) shown in the corresponding color.

**Figure 6:**
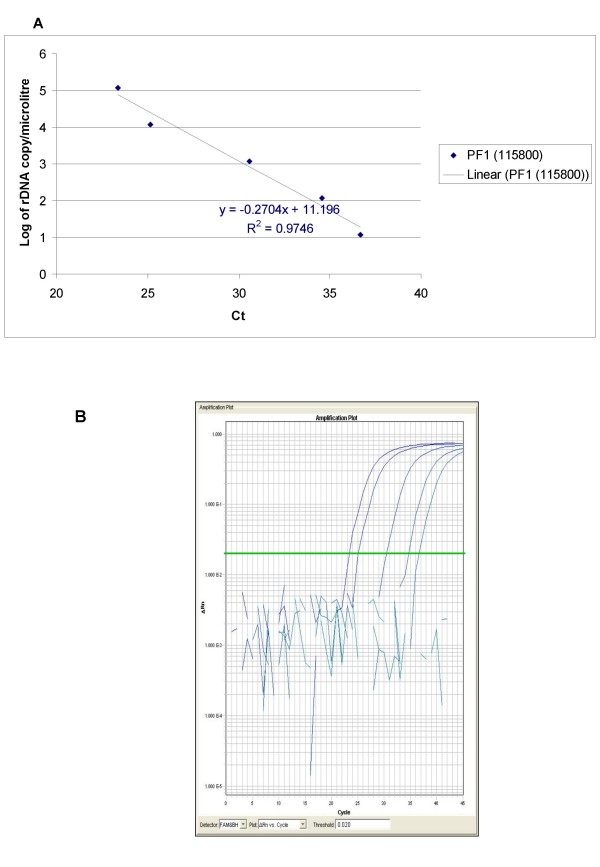
**A plot of rDNA copy number versus the threshold cycle for the 10-fold serial dilutions of *P. falciparum *specific QPCR (A) with the rDNA copy number in parentheses**. The amplification plot generated by QPCR for 10-fold serial dilutions of *P. falciparum *rDNA is also shown (B).

The ability of QPCR to be quantitative was established by the comparison between the log of parasitaemia versus Ct value (threshold cycle) for all the four species of malaria. This was evaluated on representative clinical specimens for all four species of *P. falciparum *(*n *= 3), *P. vivax *(*n *= 3), *P. malariae *(*n *= 3), and *P. ovale *(*n *= 3). An increasing parasite concentration was reflected by lower Ct value with good linear correlation for both clinical isolates of all species (Figures 1, 2, 3, 4 and 5). To establish the minimum copy number of the target rDNA gene sequence detectable by QPCR, DNA from blood specimen exclusively positive for *P. falciparum *single ring stage was used. The standard curve for QPCR was constructed by using 10-fold serial dilutions of genomic DNA (115800 to 0.01158 rDNA copies/μl). A plot of rDNA copy number versus the Ct value is shown in Figure 6A. The detection limits was found to be 11 rDNA copies/μl for *P. falciparum*. A plot of the Ct versus the log of rDNA copy/μl showed a linear correlation (R^2 ^= 0.9746). An amplification plot generated by QPCR for the 10-fold serial dilutions of *P. falciparum *rDNA is depicted in Figure 6B.

The comparative results of limit of detection showed that the QPCR had the highest sensitivity to detect parasite at lower levels (higher dilutions) as compared to microscopy and ICTs. For the three frozen biological replicates each of *P. falciparum*, *P. vivax*, *P. malariae *and *P. ovale*, there was an average log dilution difference of 2, 3, 4, and 4 respectively between QPCR and ICTs (Table 4). Similary, for the three fresh biological replicates of *P. falciparum *there was an average log dilution difference of 1 between QPCR and ICTs and an average log dilution difference of 2 between QPCR and Microscopy (Table 4). In order to understand the effect of different malarial parasite stage proportion on the QPCR detection system, a detailed evaluation of the various stages of malarial parasite and its contributing rDNA copy number for the LOD was also done (Table 5). For *P. falciparum*, detection of single ring infections required a higher rDNA copy number than double ring infections, and detection of gametocytes by QPCR required a higher rDNA copy number than for other *Plasmodium spp*. For non-*P. falciparum *species, detection of trophozoites required a higher rDNA copy number than ring stages.

**Table 4:** Comparative LOD results of Microscopy, ICTs and RTPCR performed on blood specimens from representative (*n *= 3 per species) patient specimens for each malaria species.

Species	% Parasitaemia Distribution	Microscopy	BINAXNOW	CARESTART	QPCR
***P. falciparum *1**	Parasitaemia 5.6%				
	88.2% Single Rings	1.00E-03	1.00E-04	1.00E-03	1.00E-05
	11.8% Double Rings				

***P. falciparum***	Parasitaemia 0.6%				
	100% Single Rings	1.00E-02	1.00E-03	1.00E-03	1.00E-04

***P. falciparum *3**	Parasitaemia 22.1%				
	86.8% Single Rings	1.00E-02	1.00E-04	1.00E-04	1.00E-05
	13.2% Double Rings				

***P. vivax *1**	Parasitaemia 0.474%				
	19.1% single rings	ND	1.00E-01	1.00E-02	1.00E-05
	57.4% growing trophozoites				
	19.1% mature trophozoites				
	4.2% gametocytes				

***P. vivax *2**	Parasitaemia 0.728%				
	54.7% rings	ND	1.00E-01	1.00E-02	1.00E-05
	45.3% growing trophozoites				

***P. vivax *3**	Parasitaemia 0.061%				
	70% growing trophozoites	ND	1.00E+00	1.00E-01	1.00E-03
	15% mature trophozoites				
	15% gametocytes				

***P. malariae *1**	Parasitaemia 0.06%				
	25% growing trophs	ND	1.00E+00	1.00E+00	1.00E-04
	57.1% mature trophs				
	3.6% mature schizonts				
	7.1% immature schizonts				
	7.1% gametocytes				

***P. malariae *2**	Parasitaemia 0.10%				
	11.1% rings	ND	1.00E+00	1.00E+00	1.00E-04
	50% growing trophozoites				
	13.9% mature trophozoites				
	2.8% immature schizonts				
	5.6% mature schizonts				
	16.7% gametocytes				

***P. malariae *3**	Parasitaemia 0.0614%				
	11.1% rings	ND	1.00E+00	1.00E+00	1.00E-03
	44.4% growing trophozoites				
	14.8% immature schzionts				
	11.1% mature trophozoites				
	18.5% gametocytes				

***P. ovale *1**	Parasitaemia 0.167%				
	3% rings	ND	undetectable	1.00E+00	1.00E-04
	94% growing trophozoites				
	3% mature schizonts				

***P. ovale *2**	Parasitaemia 0.219%				
	11.4% rings	ND	1.00E+00	1.00E+00	1.00E-05
	85.7% growing trophozoites				
	2.8% gametocytes				

***P. ovale *3**	Parasitaemia 0.009%				
	100% growing trophozoites	ND	undetectable	undetectable	1.00E-03

Values reflect the tenfold dilution level from the original sample.

ND- not determined due to lack of unfrozen specimens for these species.

**Table 5:** Average stage-specific detection limit of clinical specimens (*n *= 3 per species) by QPCR.

	Parasite stage specific limit of detection						
	rDNA copy number per reaction (parasites per reaction)						
	
	Single Rings	Double Rings	Growing Trophozoites	Mature Trophozoites	Gametocytes	Mature Schizonts	Immature Schizonts
***P. falciparum***	6.21 ± 2.0 (1.08 ± 0.34)	1.74 ± 1.09 (0.29 ± 0.18)			30 (5)		

***P. vivax***	3.66 ± 2.3 (0.60 ± 0.4)		4.51 ± 0.4 (0.75 ± 0.07)	21.72 (3.6)	0.590 (0.098)		

***P. malariae***	5.90 ± 4.29 (0.95 ± 0.75)		4.74 ± 2.490 (0.78 ± 0.415)	82.2 (13.7)	3.03 ± 1.7 (0.5 ± 0.3)	4.475 ± 1.9 (0.705 ± 0.2)	4.15 ± 0.94 (0.69 ± 0.16)

***P. ovale***	0.56 ± 0.18 (0.08 ± 0.02)		18 ± 4.5 (3 ± 0.75)		0.18 (0.03)	6 (1)	

LOD are stated as either ribosomal DNA copy number or parasites per reaction with standard error where multiple stages were present in specimens.

## Discussion

Performance characteristics, limit of detection, inter-reader variability and cost of QPCR, two ICTs (BINAXNOW and CARESTART) and reference microscopy was evaluated. QPCR quality assurance was sought by comparing the results of QPCR in two distinct reference lab settings. The results indicate that the QPCR is the most analytically sensitive method (sensitivity 99.41%), followed by CARESTART (sensitivity 88.24%), and BINAXNOW (sensitivity 86.47%) for the diagnosis of malaria in returning travelers when compared to microscopy. The results of the comparative LOD study between different assays suggest that the QPCR has the superior LOD. Microscopy was unable to specifically identify *Plasmodia spp*. in 18 out of 170 positive samples by QPCR. Moreover, the 17 samples that were negative by microscopy and positive by QPCR were also positive by ICTs.

Although microscopy can be sensitive to a threshold of 5-50 parasites/μl depending on the expertise of the microscopist, the average microscopist is likely to achieve a sensitivity closer to 100 parasites/μl or higher [22,23]. The possible explanation for the discordance in these samples could be the inability of microscopy to appreciate the degraded parasite, merozoites stage, sequestered parasites in the splenic vasculature, or the occult exo-erythrocytic schizogony stage. More likely, the LOD studies suggest that reference microscopy is unable to detect parasites when below a certain threshold. Interestingly, this study revealed that the limit of detection was significantly affected by the stage of malarial parasite. For example, the two *P. malariae *positive specimens "Pm1" and "Pm3" (see Table 4) had the same parasitaemia of 0.06%, but there was a marked difference of ten fold in the limit of detection. This may be due to the difference between the parasite stage proportions between the specimens. The specimen "Pm1" had higher proportion of parasite stages like growing trophozoites and mature trophozoites in comparison to "Pm3". Our QPCR appeared to be more sensitive in the detection of the malarial parasite in blood specimens than performed by our comparator institution in Alberta (see Additional file 1). This is likely because of the difference in the number of targets amplified in one tube. The QPCR screens two targets in one tube as opposed to four targets in Alberta. This suggests that the PCR reaction kinetics favours the lesser number of targets per reaction due to competition among the targets for amplification. Of note, this marginal difference did not affect result interpretation.

It has been reported that the sensitivity of ICT to detect malarial parasites decreases as the parasitaemia decreases [24]. False-negative results, particularly for *P. falciparum *with its more virulent natural history of infection, are of concern. For BINAXNOW four false-negative results occurred for *P. falciparum *infections, 13 for *P. vivax*, four for *P. ovale*, and one for *P. malariae*. For CARESTART, three false-negative results occured for *P. falciparum *infections, 10 for *P. vivax*, four for *P. ovale*, one for *P. malariae *and one for a *P. ovale *and *P. malariae *mixed infection. In most cases the false negative results were due to low parasitaemia, but in a very few cases the false negative results was even at high parasitaemia. This may be attributed to the prozone phenomenon (antibody excess) making antigen unavailable to be detected by ICT or a possible mutant gene resulting in altered HRP-2 antigen [24]. The sensitivity of CARESTART (85.2%) was slightly higher than BINAXNOW (83.67%), and both ICTs had similar specificities (99.26%). CARESTART sensitivity was higher because it was able to pick up non-*falciparum *species on more occasions than BINAXNOW, this suggests that *Plasmodium *lactate dehydrogenase (pLDH) used in CARESTART is a more sensitive marker than aldolase used in BINAXNOW for detecting non-*falciparum *species. In the majority of samples, both the ICTs have identified *P. falciparum *monoinfections (detected by microscopy and QPCR) as *P. falciparum*/mixed because both bands (*P. falciparum *and pan-malarial) were positive, thereby not allowing the technologist to rule out mixed infection on the basis of the ICT alone. The sensitivity of both ICTs for the detection of only *P. ovale *and *P. malariae *infections was 0% and 50% respectively.

Previous reports suggest that the expression levels of the *pan-Plasmodium *antigen (LDH and aldolase) in *P. ovale *and *P. malariae *may be minimal and this in combination with low parasitaemia in these infections accounts for relatively lower sensitivity for the detection of these malaria species [25,26]. It is important to note that both the ICTs have the limitation that does not distinguish between the non-*P. falciparum *species (*P. vivax*, *P. ovale*, and *P. malariae*), nor can it reliably distinguish pure *P. falciparum *infections from mixed *falciparum *infections. However, the test was found to be simple to conduct, rapid, and easy to interpret, with excellent inter-reader agreement for BINAXNOW and CARESTART as K value = 0.872 and K value = 0.898 respectively, indicating good agreement between trained and non-trained readers. Discrepancies between readers occurred mainly when the test result was weakly positive (faint band), especially in cases of low parasitaemia. Rapid diagnostic assays like ICTs may be a useful tool when expert microscopy is not available or onsite field survey is to be conducted on large population. Considering the inherent limitation and advantages of all the assays evaluated in the present study and the evidence from previous reports, ICTs are a useful diagnostic tool and can be used as an adjunct to conventional microscopy in front-line clinical laboratories in areas where malaria is not endemic. These laboratories frequently lack sufficient specimen volume to maintain expertise in diagnostic microscopy. A rapid diagnostic test like ICT could provide a fast although still preliminary diagnosis, while results are confirmed from a reference laboratory. There were three false-positive BINAXNOW results and two false-positive CARESTART results in this study. Occasional false-positive results due to the presence of rheumatoid factor have previously been reported with diagnostic methods based on the detection of HRP II [27]. It is important to note that, due to the incidental false-negative results with ICTs, malaria infection cannot be ruled out on the basis of a negative ICT result. Reference-level microscopy remains essential especially with the capability for species identification, parasite quantitation, and capacity to rule out gametocyte only infections. However, due to its excellent LOD, QPCR at the reference level serves the essential purpose of confirming or refuting microscopic speciation in certain cases (discussed below). A limitation of QPCR and ICT was that a positive result was obtained in one blood specimen with pure *P. falciparum *gametocytes. This shows that the QPCR can be positive in convalescence or clinically cured infections due to persistent sexual-stage forms (gametocytes) implying that the QPCR is unable to distinguish gametocyte only infections from true infections.

The cost and the processing time for all the four techniques employed for the laboratory diagnosis of malaria were evaluated (Table 6). At the time of writing, when purchased in bulk, the BINAXNOW kit was priced at US$5.20 per sample, whereas the CARESTART™ Malaria was priced at US$1.20 per sample. Of note, no significant difference was observed in this study between either ICT in spite of the cost differential. The BINAXNOW test has the approval from regulatory bodies in North America, such as the Food and Drug Administration and Health Canada. Reference microscopy of thick and thin smears costs US$2.50 for reagents in our reference laboratory setting. The ABI 7900HT Fast Real-Time PCR System used here for QPCR had a per sample cost of US$14.80 for reagents. Of note, QPCR requires a one time capital equipment purchase of the thermocycler. For example, the FC2500 SmartCycler System (Cepheid, Sunnyvale. CA) costs approximately US$36,000 and is capable of 14 reactions per run which seems appropriate given the frequency of malaria requests and the "stat" nature of the test in a non-endemic setting. The cost analysis clearly showed that the CARESTART was the cheapest method. While ICTs do not require trained staff, reference parasitology laboratories continue to place emphasis on microscopy training. A challenge to staffing of QPCR is the lack of equivalent training in molecular techniques. This is in contrast to virology and bacteriology sections where cross-training in molecular techniques is now common place in the reference laboratory.

**Table 6:** Comparison of the cost of different techniques employed for the diagnosis of malaria

Reagents	Microscopy	BINAXNOW	CARESTART	QPCR
**QIAmp** **DNA Extraction**	NA	NA	NA	$2.30

**TaqMan** **master mix and probe**	NA	NA	NA	$3.00

**BINAXNOW**	NA	$5.00	NA	NA

**CARESTART**	NA	NA	$1.00	NA

**Consumables**	$2.50	$0.20	$0.20	$9.50

Total cost *	$2.50	$5.20	$1.20	$14.80

**Approximate technologist time **^#^	90 mins	30 mins	30 mins	45 mins

* These are approximate cost estimates are in United States Dollars; it does not include tax and shipping

^# ^Technologist time is restricted to hands-on time with sample preparation and testing

## Conclusion

Taken together, QPCR should be implemented in all reference clinical laboratories and used in specific scenarios: (i) when a clinician does not agree with microscopic findings based on clinical pre-test probability of malaria; (ii) when two microscopists disagree on the findings particularly in cases of mixed infection, low parasitaemia, and sample degradation; (iii) when a microscopist does not visualize enough parasite stages to make a speciation call. Of note, the significance of a positive QPCR result following pharmacotherapy is still unclear as it could represent a fully treated infection with only DNA present and no live asexual stage organisms. As such, use of QPCR for test of cure is not encouraged until further studies are available. Due to the poor LOD with non-*P. falciparum *species and lack of advantage in diagnostic sensitivity when compared to reference microscopists for *P. falciparum*, the ICTs appear better suited to the community or hospital laboratory where experience with malaria smears may be limited and thus confer a diagnostic advantage.

## Abbreviations

Pf: *P. falciparum*; Pv: *P. vivax*; Pm: *P. malariae*; Po: *P. ovale*; NA: not applicable; QPCR: real-time quantitative polymerase chain reaction; ICT: immuno-chromatographic test; rDNA: ribosomal DNA; LOD: limit of detection

## Competing interests

The authors declare that they have no competing interests.

## Authors' contributions

KK carried out experimental work and co-wrote the paper; RL, DM and FR carried out experimental work; DRP provided funding, designed the study and co-wrote the paper. All authors read and approved the final manuscript.

## Supplementary Material

Additional file 1 **Comparative results of the quality assurance of QPCR on a panel of blinded blood specimens (n = 10)**. The table contains Ct values from QPCR performed in two separate reference laboratories. The data shows perfect agreement between the two centres and exemplifies the importance of a quality assurance programme for molecular diagnostics where proficiency panels are exchanged. Click here for file
