# Supplementary material for: Multiplex real-time quantitative PCR, microscopy and rapid diagnostic immuno-chromatographic tests for the detection of Plasmodium spp: performance, limit of detection analysis and quality assurance
Source: Malar J. 2009 Dec 9;8:284. doi: 10.1186/1475-2875-8-284 (PMC2796674; doi:10.1186/1475-2875-8-284)
Supplement: Additional file 1 — Comparative results of the quality assurance of QPCR on a panel of blinded blood specimens (n = 10). The table contains Ct values from QPCR performed in two separate reference laboratories. The data shows perfect agreement between the two centres and exemplifies the importance of a quality assurance programme for molecular diagnostics where proficiency panels are exchanged. [file 1475-2875-8-284-S1.DOC]

**Supplementary Tables:**

Supplementary Table 1: Comparative results of the quality assurance of QPCR on panel of blinded blood specimens (*n = 10*).

| ***Plasmodium* species** | **Ct values from AB^*^** | **Ct values from ON^#^** |
| --- | --- | --- |
| ***P. vivax*** | 26.5 | 21.6 |
| ***P. falciparum*** | 23.1 | 21.9 |
| ***P. falciparum*** | 19.1 | 18.6 |
| **Negative** | Negative | Negative |
| ***P. ovale*** | 31.0 | 27.5 |
| ***P. vivax*** | 25 | 22.1 |
| ***P. vivax*** | 29.8 | 26.6 |
| ***P. ovale*** | 27.3 | 26.6 |
| ***P. falciparum*** | 31.5 | 31.0 |
| ***P.malariae* + *P.falciparum*** | 28/38 | 29.2/35.9 |

^*^ AB - Reference provincial laboratory for public health, Edmonton, Alberta, Canada

^#^ ON- Reference provincial Toronto Public Health Laboratory, Ontario, Canada
